# Yin and Yang Regulation of Liver X Receptor α Signaling Control of Cholesterol Metabolism by Poly(ADP-ribose) polymerase 1

**DOI:** 10.7150/ijbs.50042

**Published:** 2020-09-01

**Authors:** Fengxiao Zhang, Cheng Wang, Yuhan Jiang, Kun Huang, Fangmei Liu, Meng Du, Xi Luo, Dan Huang, Kai Huang

**Affiliations:** 1 Department of Cardiovascular Diseases, Union Hospital, Tongji Medical College, Huazhong University of Science and Technology.; 2 Clinical Center for Human Genomic Research, Union Hospital, Huazhong University of Science and Technology.

**Keywords:** PARP1, LXRα, cholesterol metabolism

## Abstract

Liver X receptor α (LXRα) controls a set of key genes involved in cholesterol metabolism. However, the molecular mechanism of this regulation remains unknown. The regulatory role of poly(ADP-ribose) polymerase 1 (PARP1) in cholesterol metabolism in the liver was examined. Activation of PARP1 in the liver suppressed LXRα sensing and prevented upregulation of genes involved in HCD-induced cholesterol disposal. Mechanistically, LXRα was poly(ADP-ribosyl)ated by activated PARP1, which decreased DNA binding capacity of LXRα, thus preventing its recruitment to the target promoter. Intriguingly, we found that unactivated PARP1 was indispensable for LXRα transactivation and target expression. Further exploration identified unactivated PARP1 as an essential component of the LXRα-promoter complex. Taken together, the results indicate that activated PARP1 suppresses LXRα activation through poly(ADP-ribosyl)ation, while unactivated PARP1 promotes LXRα activation through physical interaction. PARP1 is a pivotal regulator of LXRα signaling and cholesterol metabolism in the liver.

## Introduction

Nuclear receptors (NRs) comprise a family of ligand-inducible transcription factors involved in metabolism, development and reproduction 1. The liver X receptors LXRα (NR1H3) and LXRβ (NR1H2) are oxysterol-activated transcription factors belonging to the steroid/thyroid hormone nuclear receptor superfamily 2-4. Upon activation, the LXRs form an obligate heterodimer with retinoid X receptor (RXR) and subsequently bind to LXR response elements (LXREs) in the target promoter to regulate gene expression 5. Of these 2 isoforms, LXRα is the master regulator of cholesterol homeostasis through its regulation of genes involved in cholesterol metabolism 6-10. LXRα signaling has been implicated in the pathogenesis of steatohepatitis and hypercholesterolemia 11. Efforts to clarify the molecular mechanisms controlling LXRα activity could provide new insights into potential strategies to treat disorders of liver cholesterol metabolism.

Poly(ADP-ribose) polymerase (PARP) 1 accounts for approximately 90 percent of cellular PARP activity 12-15. Upon activation, it catalyzes the transfer of ADP-ribose units from NAD^+^ onto acceptor proteins 12-15. This process, namely poly(ADP-ribosyl)ation is an important post-translational modification of proteins, by which PARP1 modifies the properties and functions of acceptor proteins, in addition to being involved in multiple cellular processes, such as DNA repair and replication, transcription, chromatin remodeling etc. 16. Previous studies have revealed that PARP1-mediated poly(ADP-ribosyl)ation can regulate the activities of transcription factors, such as PARP1γ, FXRα, ERα and AP-1 17-20. Recently, studies have shown that inhibition of PARP1 alleviates hypercholesterolemia and steatohepatitis in mice, implicating a potential role of PARP1 in the pathogenesis of cholesterol disorders 21.

In this study, we reveal a physical and functional coupling between PARP1 and LXRα in the suppression of hepatic cholesterol disposal in HCD-treated mice. PAPR1 exerts two opposite effects on LXRα signaling: activated PARP1 suppresses LXRα transactivity through poly(ADP-ribosyl)ation whereas unactivated PARP1 increases LXRα transactivity through physical interaction.

## Methods

### Ethics Statement

All animal experiments in this study conformed to the National Institutes of Health (NIH) Guide for the Care and Use of Laboratory Animals and were approved by the Ethics Committee of Tongji Medical College, Huazhong University of Science and Technology, under permit number “[2010] S058”.

### Animal models

8-10 weeks old male C57BL/6 wildtype mice, 129Sv PARP1 knock out (PARP1^-/-^, PKO) mice and C57BL/6 LXRα knock out (LXRα^-/-^, LKO) mice were obtained from Jackson Laboratory. We crossed mice of 129Sv PKO mice to generate C57BL/6 background PKO mice. All mice were housed in specific pathogen free animal house at Huazhong University of Science and Technology. All animal studies were performed in adherence with the Guide for the Care and Use of Laboratory Animals published by the NIH and approved by The Institutional Animal Care and Use Committee of Huazhong University of Science and Technology.

C57BL/6 background PKO or WT mice were randomly divided into four groups, depending on diet, which each animal received for 28 days: (1) Chow group (n=8): animals were fed with standard chow diet (SCD) (8% rice bran, 51% maize, 30% soybean powder, 3% bone powder, 1.3% multivitamin and 6.7% mineral); (2, 3 & 4) Animals were fed a high cholesterol diet (HCD) (79.5% SCD, 3% cholesterol, 7% lard, 10% yolk powder and 0.5% bile salt). From day 15 for a further 2 weeks they were intraperitoneally injected once a day with either 3-aminobenzamide (3AB, 30mg/kg/d, Sigma, n=8, HCD+3AB group), N-(6-oxo-5, 6-dihydrophenanthridin-2-yl)-2-(N, N-dimethylamino)acetamide (PJ34, 10 mg/kg/day, Sigma, n=8, HCD+PJ34 group), or an identical volume of normal saline as a benign vehicle (n=8, HCD+NS group).

C57-WT or LKO mice (either male or female) were randomly divided into three groups, depending on diet, which each animal received for 28 days, as follows: (1) Chow group: C57-WT or LKO mice fed a SCD; (2) HCD group: C57-WT or LKO mice fed a HCD; (3) HCD+PJ34 group: from day 15, HCD animals were intraperitoneally injected with PJ34 (10 mg/kg/day, Sigma) once per day for two weeks.

Eight to ten week old PKO mice (either male or female) were fed a SCD (chow) or HCD for 4 weeks with injection at days 7 and 20 through a tail-vein of a recombinant adenovirus (1×10^9^ pfu) encoding wild type human PARP1 gene (HCD+wt-PARP1) or an enzymatically inactive mutant (mut) PARP1 (HCD+mut-PARP1) or vector (HCD+vector). At day 28, all mice were fasted for 16 hours prior to being sacrificed. Transfection efficiency was determined by real time RT-PCR.

Serum samples were drawn from each mouse from the eye socket vein at days 0, 14, 28 for serum biochemical analysis. At day 28, all animals were anesthetized with pentobarbital (50mg/kg, Sigma, Burlington, MA, USA). The livers were excised and immediately weighed.

### Tissue chemistry

Cholesterol of liver tissue was extracted as described previously study 22. Plasma HDL, LDL, cholesterol and hepatic cholesterol levels were determined using a commercial kit (Wako Pure Chemical Industries, Osaka, Japan). The bile acid pool size was determined using a Wako total bile acid test (Wako Pure Chemical Industries, Osaka, Japan) following ethanol extraction from a mixed tissue sample comprising liver, gallbladder and the whole small intestine 23.

Mevalonic acid was quantified by liquid chromatography/electrospray ionization tandem mass spectrometry (LC/ESI/MS/MS), using positive-ion mode electrospray ionization with a 5500 Q-Trap system (Applied Biosystems, Foster City, CA) as described previously 24, 25.

### Cell Cultures

Primary mice hepatocytes were isolated from mice, as previously described 26. Human hepatoma HepG2 cells (American Type Culture Collection, Manassas, VA, USA) were purchased from the Cell Bank of Type Culture Collection of the Chinese Academy of Sciences. Cells were maintained in 1640 medium (Gibco, Thermo Fisher Scientific, Waltham, Massachusetts, USA), and then incubated with either 3AB for 24h, PJ34 for 24h, hydrogen peroxide (for 30 mins, GW3965 for 24h or the appropriate vehicle for processing for RNA isolation, Western blotting or EMSA assay. 3AB, PJ34, hydrogen peroxide and GW3965 were obtained from Sigma (Burlington, MA, USA).

### DNA Manipulation and Plasmids

The mammalian expression vectors pCDNA3.1-flag-PARP1 encoding wild-type PARP1 was a gift from Dr. Yun Zhang (Qilu Hospital, Shandong University, China). The mut-PARP1 plasmids in which lysine 893 had been replaced with isoleucine (K893I) was generated as described previously using a QuikChange site-directed mutagenesis kit (agilent technologies, Santa Clara, California, USA). Primer: 5'-CAGGCTACATGTTTGGTATAGGGATCTATTTCGCTGAC-3' 5'-TCACGGGCGCTTCAGGCGGG-3'.

The luciferase-reporter plasmid LXRE-TK-LUC (containing three copies of the LXRE consensus sequence) and control plasmid TK-LUC were kindly provided by Dr. David J. Mangelsdorf (University of Texas Southwestern Medical Center, USA) 27. Wild type and mutant ABCA1 promoter (980 bp) plasmids (PGL3-WT-ABCA1 and PGL3-DR-ABCA1) were kindly provided by Dr. Yoshinari Uehara (Department of Cardiology, Faculty of Medicine, Fukuoka University, Japan) 28 in which a mutation was created at the DR4 site: TGACCGATAGTAACCT→TGUTGTUGATAGT AUCTAUT.

### Electrophoretic Mobility Shift Assay (EMSA) and Supershift Assay

DNA-protein interactions were detected using a LightShift^TM^ Chemiluminescent EMSA kit (Pierce, Thermo Fisher Scientific, Waltham, Massachusetts, USA) according to the manufacturer's protocol. The LXRE consensus oligonucleotide sequences were: forward: 5'-CAAGGATGTGTCCCTTCAACTCAATGTGGC-3'; reverse: 5'- GCCACATTGAGTTGAAGGGACACATCCTTG-3'. Each 5' end was labeled with biotin. In the supershift assay, after incubation of the nuclear extracts with 2µg appropriate antibody or IgG at 4°C for 60 minutes, biotin-labeled oligonucleotides were added to the reaction and incubated for further EMSA assay.

### Chromatin immunoprecipitation (ChIP) and re-ChIP assay

ChIP assays were performed as previously described 29. Hepatocytes were sonicated and lysates were immunoprecipitated with appropriate antibody or IgG (negative control). In re-ChIP assays, chromatin was immunoprecipitated with anti-LXRα antibody, eluted with elution buffer supplemented with 10 mM DTT at 37°C for 30 min, then diluted 25 fold with dilution buffer (20 mM Tris-HCl (pH 8.0), 150 mM NaCl, 2 mM EDTA, 1% Triton X-100), and finally re-immunoprecipitated with IgG or an antibody against PARP1 or PAR (Santa Cruz Biotechnology, Santa Cruz, California, USA). Realtime-PCR was performed using 1 μg of template DNA with specific primers for human ABCG1: sense primer: TCAGGATCTGGATGGTGAATG; antisense primer: CACAGTGGGGAAGTAAGGCA. Input chromosomal DNA and ChIP DNA with non-specific IgG were subjected as negative control.

### RNA interference and transfection

Small interfering RNA (siRNAs) was synthesized by RiBoBio Co. Ltd (Guangzhou, Guangdong, China). Transfection of siRNA was performed at a final concentration of 50 nM using Lipofectamine 2000 (Invitrogen, Carlsbad, California, USA). The siRNA sequences are shown in Table 1.

### Preparation of whole and nuclear extracts

The methods for the preparation of whole cell and nuclear extracts have been described previously 18, 20. Protein concentrations of these extracts were determined using Bradford assay. The cell extracts obtained using this method was stored at -80°C until required for analysis.

### Real Time RT-RCR Assay

Total RNA was isolated using Trizol (Takara Bio, Gunma, Oizumi, Japan) according to the manufacturer's instructions. One μg of total RNA was reverse transcribed using RNA-PCR Kit (Takara Bio, Gunma, Oizumi, Japan) and the resulting cDNA used as PCR template. The mRNA levels were determined by real-time PCR using ABI PRISM 7900 Sequence Detector system (Applied Biosystems, Thermo Fisher Scientific, Waltham, Massachusetts, USA) according to the manufacturer's instructions.

### PARP activity assay

PARP activity was assayed using universal colorimetric PARP assay kit (Trevigen, Helgerman CT, Gaithersburg. USA) according to the manufacturer's instructions. Cell lysates containing 50 μg protein were loaded into 96-well plate coated with histones and biotinylated poly ADP-ribose and incubated for 1 hour, treated with strep-HRP, then read at 450 nm in a spectrophotometer.

### Western Blot Assay and *in vitro* protein-protein interaction assay (far-Western blot)

Western blot analysis was performed as previously described 30. Antibodies against PARP1 (R&D, McKinley Place NE Minneapolis, USA), PAR (Trevigen, Helgerman CT, Gaithersburg. USA), histone H1 (Santa Cruz Biotechnology, Santa Cruz, California, USA), β-actin (Santa Cruz Biotechnology, Santa Cruz, California, USA), GAPDH (Santa Cruz Biotechnology, Santa Cruz, California, USA) or LXRα (Abcam, Cambridge, England) were used as primary antibodies. Specific bands detected using a chemiluminescence assay (ECL detection reagents, Pierce, USA) were recorded onto X-ray film. BioRad Quantity One software (version 4.4) was used for quantification.

Far-western blot assays and AP-PARP1 protein were performed as described previously 17. Membranes were incubated with 1µg/ml recombinant PARP1 protein (Trevigen, Helgerman CT, Gaithersburg. USA) or 1 µg/ml AP-PARP1 protein, 1 µg/ml recombinant protein LXRα (Abnova, Taiwan) or 1 µg/ml recombinant β-actin (Abnova, Taiwan).

### Immunoprecipitation assay

Briefly, 500 µg of nuclear extract were incubated with an antibody against PARP1, LXRα, PAR or nonspecific IgG (negative control) at 4°C for 1 hour, then on protein-G agarose at 4°C for 12 hours. The immunoprecipitants were pelleted by centrifugation at 5000×g for 1-minute then washed 4 times with lysis buffer. The pellets were suspended in SDS gel loading buffer, boiled for 10 mins, and analyzed using Western blot analysis.

### Statistical analysis

Values are shown as means ± SEM (n≥3). The significance of differences was estimated using the independent samples T test or one-way ANOVA followed by Student-Newmann-Keuls multiple comparison test. *P*<0.05 was considered significant. All statistical analyses were performed using SPSS software (version 11.0, SPSS Inc).

## Results

### Inhibition of PARP1 activation alleviated HCD-induced hepatic cholesterol accumulation through activation of the LXRα pathway

**Figure 1:**
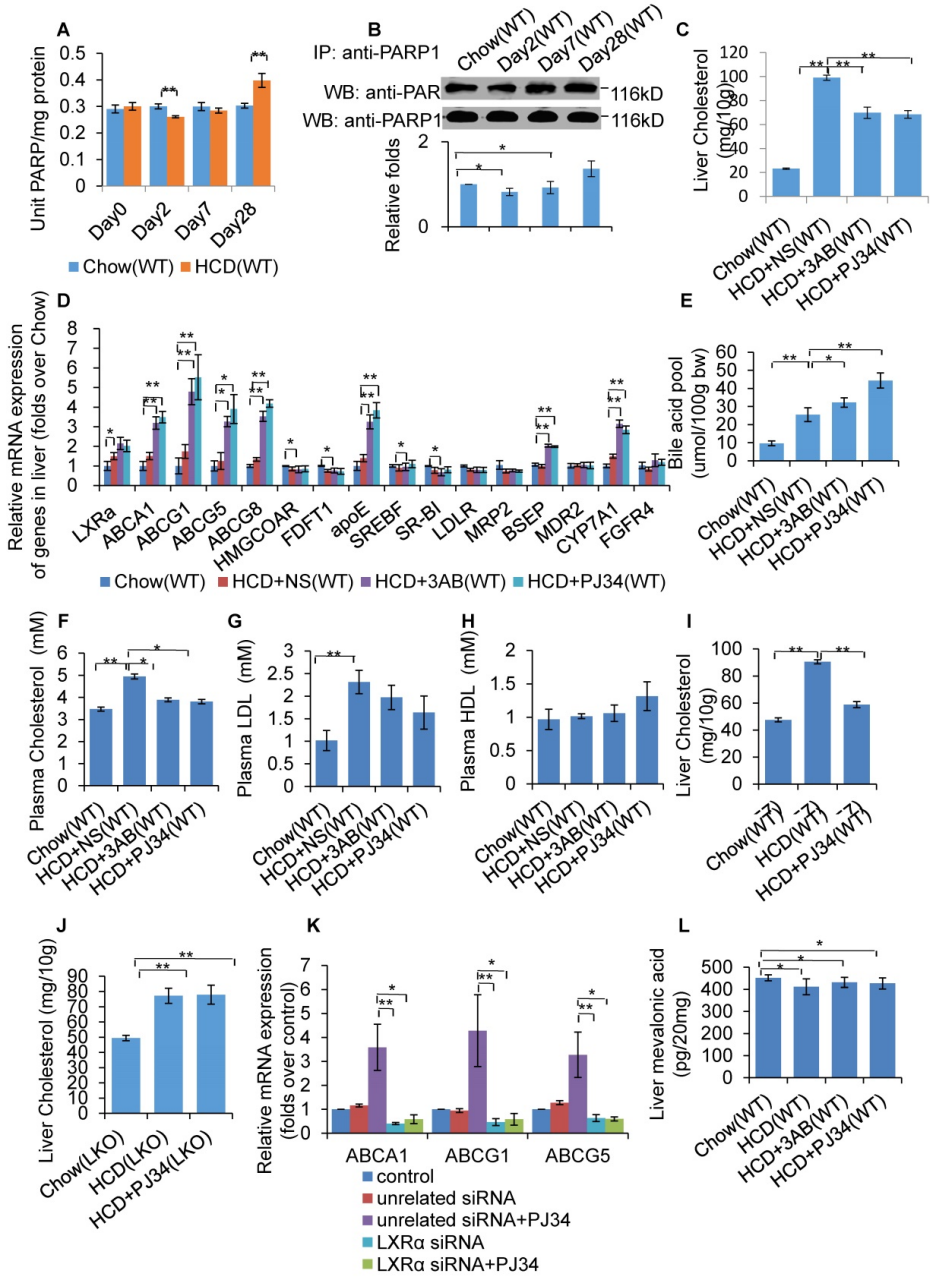
Inhibition of PARP1 activation alleviated HCD-induced hepatic cholesterol accumulation through activation of the LXRα pathway. In A-B, WT mice were treated with SCD (Chow group, n=8) or HCD (HCD group, n=8). Data are expressed as the mean ± SEM. ^**^P<0.01 vs SCD (Chow) group. (A) PARP activity in liver of WT mice. (B) Poly(ADP-ribosyl)ation levels of PARP1 in whole extracts of hepatocytes from WT mice treated with SCD or HCD, determined by IP with PARP1 antibody, followed by Western blot analysis using anti-PAR antibody. In C-F and J, WT mice were treated with SCD (Chow group, n=8), or HCD combined with intraperitoneal injection of 3AB (30mg/kg/d, HCD+3AB group, n=8), PJ34 (10 mg/kg/day, HCD+PJ34 group, n=8) or an identical volume of normal saline (NS, HCD+NS group, n=8) once per day. (C) Cholesterol concentration in mouse livers. (D) mRNA expression of selected genes in WT mice. (E) Bile acid pool size in WT mice. (F) Plasma cholesterol concentration of WT mice. (G) Plasma LDL concentration of WT mice. (H) Plasma HDL concentration of WT mice. (I) Liver cholesterol concentration in C57 mice which had been fed either a SCD (Chow group, n=8), or HCD (n=8) combined with an intraperitoneal injection of PJ34 (10 mg/kg/day, HCD+PJ34 group, n=8) once per day. (J) Liver cholesterol concentration in LKO mice which had been fed either a SCD (Chow group, n=8), or HCD (n=8) combined with an intraperitoneal injection of PJ34 (10 mg/kg/day, HCD+PJ34 group, n=8) once per day. (K) mRNA expression of selected genes in HepG2 cells which had been pretreated with LXRα siRNA (50 nM, 48h) or unrelated siRNA (50 nM, 48h), followed by treatment with PJ34 (10 μM) for 24 hours. (n=3) (L) Mevalonic acid concentration in mouse livers. In A and C-L, data are expressed as the mean ± SEM, **P<0.01, *P<0.05.

WT mice were fed a HCD or SCD for 4 weeks, and then hepatic PARP activity was determined. As shown in Figure 1A, HCD mice exhibited reduced PARP activity from day 2, but increased PARP activity at day 28, indicating that HCD feeding affected hepatic PARP activity. Furthermore, nuclear PARP1 from the mouse liver, and poly(ADP-ribosyl)ation levels of PARP1 were determined. Consistent with the PARP activity, poly(ADP-ribosyl)ation of PARP1 was suppressed at day 2, but increased at day 28 (Figure 1B). These results indicate that short term HCD feeding suppressed liver PARP1 activation, while chronic HCD feeding promoted.

To investigate whether PARP1 was involved in HCD-induced hepatic cholesterol accumulation, HCD fed mice were treated with 2 structurally unrelated PARP inhibitors, 3AB or PJ34. Results demonstrated that 3AB or PJ34 treatment decreased hepatic cholesterol content (Figure 1C), indicating that inhibition of PARP1 suppressed HCD-induced cholesterol accumulation. Realtime-PCR assays also showed that 3AB or PJ34 treatment increased the expression of genes involved in cholesterol catabolism and efflux pathway, including CYP7a1, ABCA1, ABCG1, ABCG5, ABCG8, BSEP and ApoE, while there was no significant change in expression of other target genes (Figure 1D). Consistent with this, we found that 3AB or PJ34 increased the bile acid pool size and the quantity of metabolites of cholesterol catabolism in HCD mice (Figure 1E). Furthermore, HCD-induced hypercholesterolemia was also alleviated after 3AB or PJ34 treatment (Figures 1F, 1G & 1H).

In the liver, LXRα senses the loading of dietary cholesterol and in response, increases the expression of genes involved in cholesterol catabolism and efflux pathway to facilitate cholesterol disposal. Chronic intake of HCD leads to hepatic cholesterol over-accumulation and hypercholesterolemia, reflecting insufficient activation of LXRα signaling 7, 31, 32. As LXRα is a master regulator controlling cholesterol disposal-related gene expression, we then examined whether the protective effects of PJ34 were mediated through LXRα. To confirm this hypothesis, LKO and C57-WT mice that were fed a HCD were treated with PJ34. As shown in Figures 1I and 1J, PJ34 treatment upregulated cholesterol disposal-related gene expression and suppressed liver cholesterol accumulation in WT mice, but not in LKO mice, indicating that LXRα indeed mediated the effects of PARP1 inhibition. In line with this finding, depletion of LXRα also negated the PJ34-induced increase in ABCA1, ABCG1 and ApoE gene expression in HepG2 cells (Figure 1K).

In this study, we additionally examined the effects of PJ34 and 3AB on liver cholesterol synthesis. The LC-MS/MS assay revealed that neither PJ34 nor 3AB treatment modified the hepatic content of mevalonic acid (Figure 1L). Moreover, the effect of 3AB or PJ34 treatment on the expression of genes involved in cholesterol synthesis, including HMG-CoA reductase and FDFT1 (Figure 1D). Therefore, inhibition of PARP1 did not alter hepatic cholesterol synthesis.

### LXRα was a substrate of PARP1-mediated poly(ADP-ribosyl)ation reaction in hepatocytes

**Figure 2:**
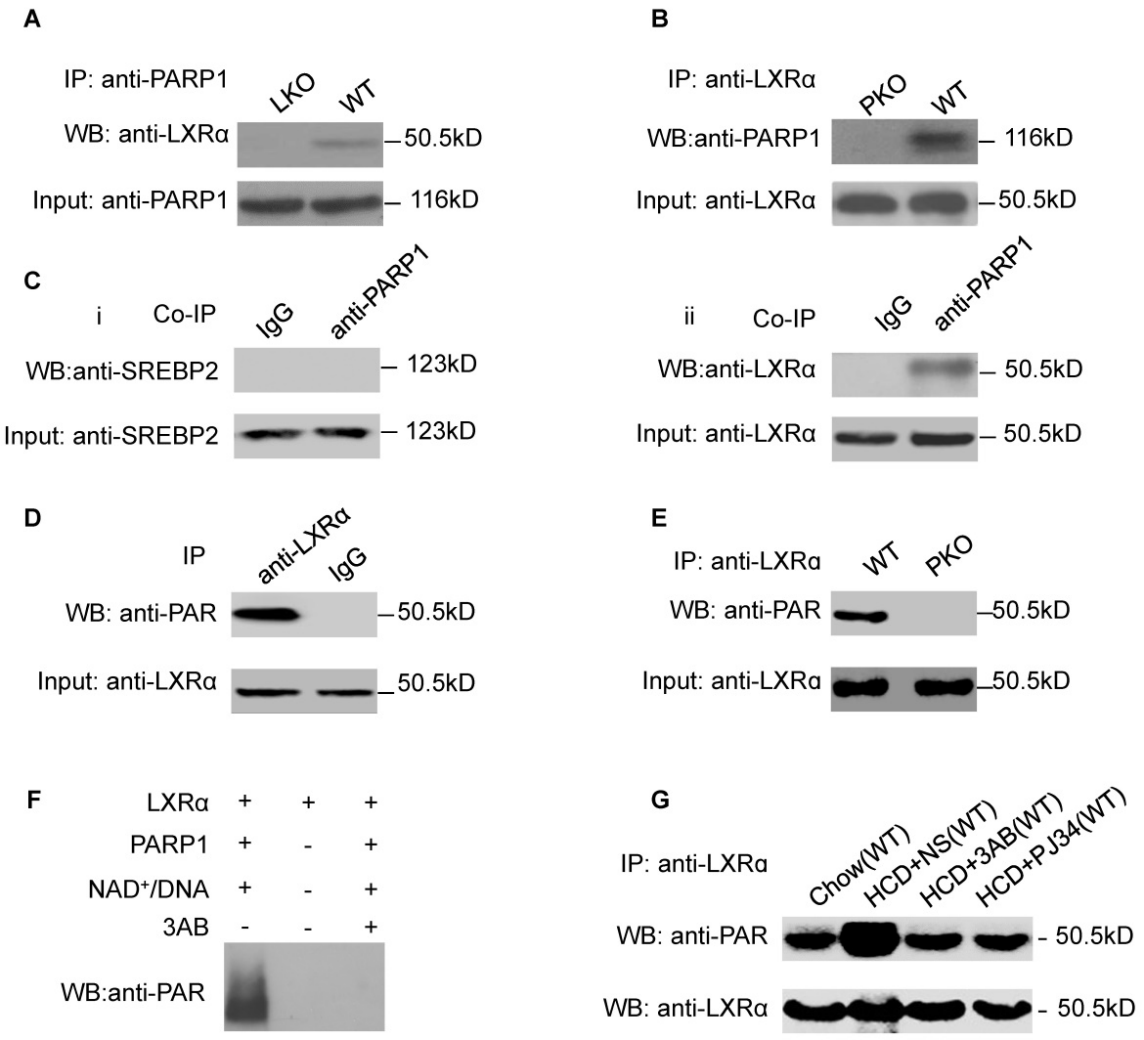
LXRα was a substrate of PARP1-mediated poly(ADP-ribosyl)ation reaction in hepatocytes. (A) Co-immunoprecipitation (Co-IP) of PARP1 bound proteins from the hepatocytes of C57-WT or LKO mice, followed by Western blot analysis using an anti-LXRα antibody (n=3). (B) Co-immunoprecipitation (Co-IP) of PARP1-bound proteins from hepatocytes of WT or PKO mice, followed by Western blot analysis using an anti-LXRα antibody (n=3). (C) Co-immunoprecipitation (Co-IP) of PARP1-bound proteins from HepG2 cells, followed by Western blot analysis using either (i) SREBP2 or (ii) LXRα antibodies. Non-specific IgG served as negative control (n=3). (D) Poly(ADP-ribosyl)ation levels of LXRα in whole extracts of HepG2 cells determined by IP with LXRα, followed by Western blot analysis using anti-PAR antibody. Non-specific IgG served as negative control (n=3). (E) Poly(ADP-ribosyl)ation levels of LXRα in whole extracts of hepatocytes from WT and PKO mice determined by IP with LXRα, followed by Western blot analysis using anti-PAR antibody. Western-blotting with anti-LXRα antibody served as the loading control (n=3). (F) Recombinant LXRα proteins incubated with PBS vehicle, PARP1/NAD^+^/active DNA or PARP1/NAD^+^/active DNA/3AB. Western blot analysis was used to detect LXRα poly(ADP-ribosyl)ation levels (n=3). (G) LXRα poly(ADP-ribosyl)ation levels in the livers of WT mice determined by IP followed by Western blot analysis using anti-PAR antibody. WT mice were fed SCD (n=8), or HCD combined with NS (n=8), 3AB (n=8) or PJ34 (n=8).

As we had established that LXRα mediates the effects of PARP1 inhibition on hepatic cholesterol metabolism, we wished to explore the interaction of PARP1 with LXRα. Nuclear extracts from hepatocytes of C57-WT or LKO mice were assayed by co-immunoprecipitation (co-IP) with an anti-PARP1 antibody. As shown in Figure 2A, nuclear LXRα could be precipitated by the anti-PARP1 antibody from C57-WT hepatocytes, but not from LKO hepatocytes, indicating that a physical interaction occurs between LXRα and PARP1. Concomitant with this conclusion, nuclear PARP1 could also be precipitated by anti-LXRα antibody from WT hepatocytes, but not from PKO hepatocytes (Figure 2B). In this study, we also examined whether PARP1 could interact with SREBP2, another cholesterol metabolism-related transcription factor. However, in contrast to LXRα, SREBP2 could not be precipitated by the anti-PARP1 antibody (Figure 2C).

We next explored whether LXRα could be poly(ADP-ribosyl)ated by PARP1. Nuclear LXRα was precipitated by anti-LXRα antibody then analyzed by Western blotting with an anti-PAR antibody (Figure 2D). As shown in Figure 2E, poly(ADP-ribosyl)ation of LXRα was detected in nuclear extracts of WT hepatocytes, but not of PKO hepatocytes, indicating that LXRα could be poly(ADP-ribosyl)ated by PARP1. This finding was further confirmed by the observation that incubation of LXRα with recombinant PARP1 and NAD^+^/nicked DNA resulted in poly(ADP-ribosyl)ation of LXRα in a cell-free system (Figure 2F). Furthermore, HCD mice displayed increased poly(ADP-ribosyl)ation of LXRα (Figure 2G). All these suggested that chronic HCD feeding promotes poly(ADP-ribosyl)ation of liver LXRα.

### Poly(ADP-ribosyl)ation prevented LXRα transactivation and target expression

**Figure 3:**
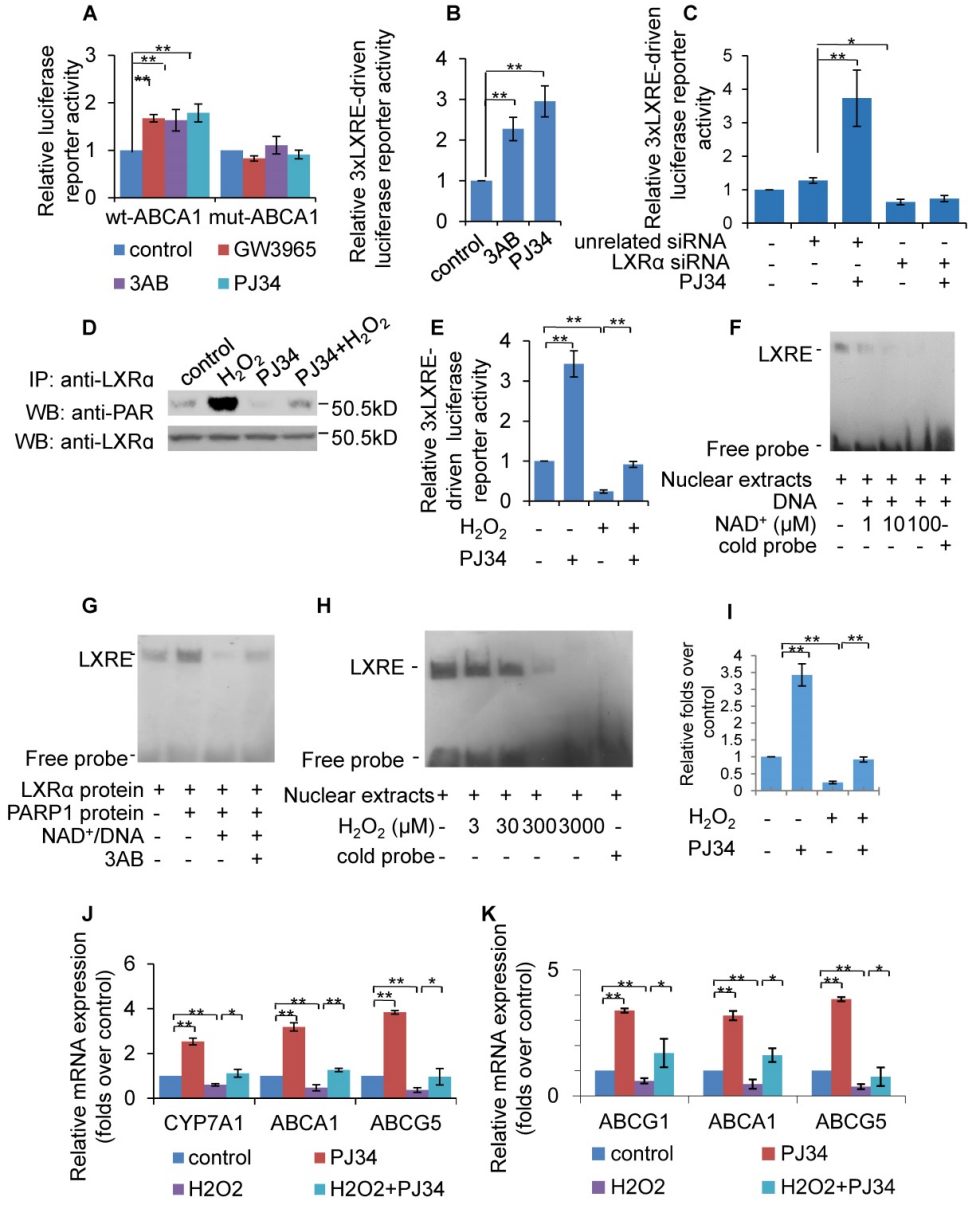
Poly(ADP-ribosyl)ation prevented LXRα transactivation and target expression. (A) Relative luciferase activity of HepG2 cells transfected with ABCA1 promoter-driven luciferase reporter. Wild-type human ABCA1 promoter (WT-ABCA1) and mutant (DR-ABCA1) were co-transfected with pRL-SV40 plasmid. Cells were treated with vehicle (PBS), GW3965 (1μM, 24h), 3AB (7mM, 24h) or PJ34 (5μM, 24h) (n=3). (B) Relative luciferase activity of HepG2 cells transfected with LXRE-driven luciferase reporter. Cells were treated with vehicle (PBS), 3AB (7mM, 24h) or PJ34 (5μM, 24h) (n=3). (C) Relative luciferase activity of HepG2 cells transfected with LXRE-driven luciferase reporter. Cells were pretreated with LXRα siRNA (50 nM, 48h) or unrelated siRNA (50 nM, 48h), followed by treatment with PJ34 (10 μM) for 24 hours (n=3). (D) Poly(ADP-ribosyl)ation levels of LXRα in whole extracts of HepG2 cells were quantified using IP with LXRα, followed by Western blot analysis using anti-PAR antibody. Western-blotting with anti-LXRα antibody served as the loading control. Cells were treated with vehicle (PBS) or H_2_O_2_ (0.3 mM, 0.5h) in the absence or presence of PJ34 (10 μM, 24h) (n=3). (E) Relative luciferase activity of HepG2 cells transfected with LXRE-driven luciferase reporter. Cells were treated with vehicle (PBS) or H_2_O_2_ (0.3 mM, 0.5h) in the absence or presence of PJ34 (10 μM, 24h) (n=3). (F) Nuclear extracts from non-treated HepG2 cells were incubated with active DNA and NAD^+^ (1, 10, 100 μM), then analyzed using an EMSA assay (n=3). (G) EMSA assay of recombinant proteins in cell free system using LXRE as probe. Recombinant LXRα protein was incubated with recombinant PARP1 protein, PARP1 protein/NAD^+^/active DNA or PARP1 protein/NAD^+^/active DNA/3AB (n=3). (H) EMSA assay of LXRα-LXRE complex formation in nuclear extracts from HepG2 cells treated with vehicle (PBS) or H_2_O_2_ (3, 30, 300, 3000 μM, 0.5h) (n=3). (I) ChIP-PCR assay using anti-LXRα antibody for amplification of ABCG1 promoters in HepG2 cells following treatment with vehicle (PBS) or H_2_O_2_ (0.3 mM, 0.5h) in the absence or presence of PJ34 (10 μM, 24h) (n=3). (J) mRNA expression of selected genes in primary hepatocytes from WT mice. Cells were treated with vehicle (PBS) or H_2_O_2_ (0.3 mM, 0.5h) in the absence or presence of PJ34 (10 μM, 24h) (n=3). (K) mRNA expression of selected genes in HepG2 cells treated with vehicle (PBS) or H_2_O_2_ (0.3 mM, 0.5h) in the absence or presence of PJ34 (10 μM, 24h) (n=3). In A-C, E and I-K, data are expressed as mean ± SEM, ^**^P<0.01, ^*^P<0.05.

To evaluate the effect of PARP1 inhibition on LXRα transactivation, luciferase reporters driven by wild-type (wt) and DR4 mutant (direct repeat with four intervening nucleotides in LXRE) promoters of the human *ABCA1* gene were transfected into HepG2 cells, and their respective luciferase activity examined thereafter. As shown in Figure 3A, treatment with PJ34, 3AB or the LXRα agonist GW3965 increased the luciferase activity driven by the wt-promoter, but not by the DR4 mut-promoter. To further confirm this finding, the effects of PARP inhibitors on luciferase reporter driven by 3xLXRE were evaluated. As shown in Figure 3B, both PJ34 and 3AB treatment of cells resulted in increased luciferase activity driven by 3xLXRE, implicating that the inhibition of PARP1 promoted LXRα transactivation. Concomitantly, knockdown of LXRα abolished PJ34-induced 3xLXRE activation (Figure 3C).

Furthermore, cells were challenged with hydrogen peroxide (H_2_O_2_), a well-established activator of PARP1 32. Treatment with H_2_O_2_ not only increased the poly(ADP-ribosyl)ation of LXRα, but also suppressed 3xLXRE-driven luciferase activity, indicating that poly(ADP-ribosyl)ation suppressed LXRα transactivation (Figures 3D & 3E). In support of this finding, administration of PJ34 significantly reversed H_2_O_2_-induced inhibition of 3xLXRE-driven luciferase activity (Figure 3E). besides, EMSA assay uncovered that, incubation of nuclear extracts from HepG2 cells with NAD^+^/nicked DNA, which promoted endogenous LXRα poly(ADP-ribosyl)ation, decreased the LXRα-LXRE complex formation (Figure 3F). Consistently, incubation of LXRα with recombinant human PARP1 and NAD^+^/nicked DNA effectively suppressed LXRα-LXRE complex formation in a cell free system (Figure 3G). In line with this finding, treatment with H_2_O_2_ reduced LXRα-LXRE complex formation in a dosage-dependent manner (Figure 3H). These results indicate that poly(ADP-ribosyl)ation reduced the DNA binding capacity of LXRα.

We then explored whether poly(ADP-ribosyl)ation decreased the recruitment of LXRα to the target promoter. ChIP assay showed that H_2_O_2_ treatment suppressed recruitment of LXRα to the ABCG1 promoter, and this inhibition was reversed by administration of PJ34 (Figure 3I). Consistent with this, H_2_O_2_ treatment also inhibited ABCA1, ABCG1 and ABCG5 expression (Figures 3J & 3K). Taken together, these results demonstrate that poly(ADP-ribosyl)ation can suppress LXRα binding to its target promoter, leading to decreased expression of its target genes.

### PARP1 is required for LXRα transactivation and target expression

**Figure 4:**
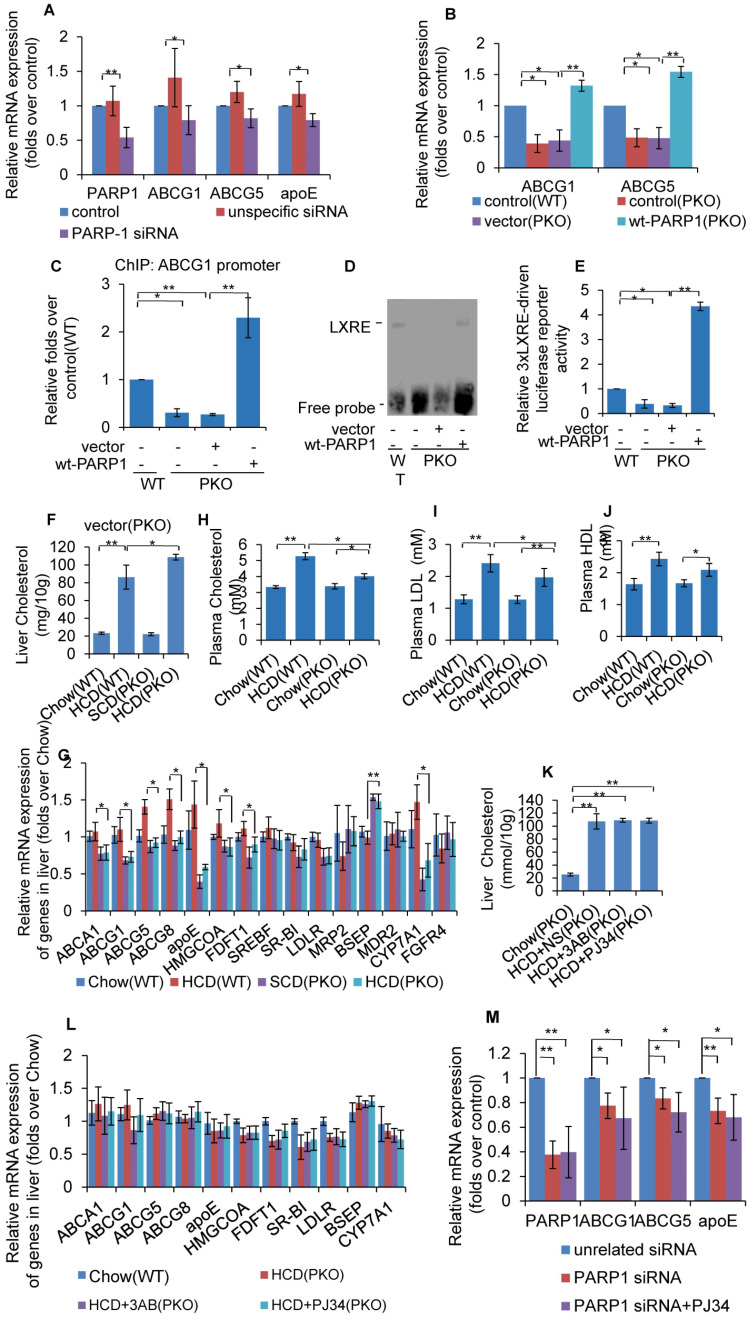
Un-poly(ADP-ribosyl)ated PARP1 is required for LXRα transactivation and target expression. (A) mRNA expression of selected genes in HepG2 cells treated with PARP1 siRNA (50 nM, 48h) or unrelated siRNA (50 nM, 48h) (n=3). (B) mRNA expression of selected genes in hepatocytes from WT or PKO mice transfected with either empty (pCDNA3.1) or full-length (wt-PARP1) vector for 48 hours (n=3). (C) ChIP-PCR assay using anti-LXRα antibody for amplification of ABCG1 promoters in hepatocytes from WT or PKO mice. Cells were transfected with empty or full-length vector (wt-PARP1) (n=3). (D) EMSA assay of LXRα-LXRE complex formation in hepatocytes from WT or PKO mice transfected with empty (pCDNA3.1) or full-length (wt-PARP1) vector for 48 hours (n=3). (E) Relative LXRE-driven luciferase activity in hepatocytes from WT or PKO mice. Cells were transfected with empty or full-length vector (wt-PARP1) (n=3). In F-G, WT or PKO mice were fed a SCD (Chow group, n=8) or HCD (n=8). (F) Cholesterol concentration in the livers of WT and PKO mice. (G) mRNA expression of selected genes in the livers of WT and PKO mice assessed by real time RT-PCR assay. In H-I, PKO mice were fed a SCD Chow group, n=8), or HCD combined with intraperitoneal injection with AB (30mg/kg/d, HCD+3AB group, n=5), PJ34 (10 mg/kg/day, HCD+PJ34 group, n=5) or an identical volume of normal saline (NS, HCD+NS group, n=5), once per day. (H) Plasma cholesterol concentration in WT or PKO mice. (I) Plasma LDL concentration in WT or PKO mice. (J) Plasma HDL concentration in WT or PKO mice. (K) Cholesterol concentration in the livers of PKO mice. (L) mRNA expression of selected genes in PKO mice. (M) mRNA expression of selected genes in HepG2 cells treated with PARP1 siRNA (50 nM, 48h) or unrelated siRNA (50 nM, 48h) in the absence or presence of PJ34 (10 μM, 24h) (n=3). In A-C and E-M, data repesent mean ± SEM, *P<0.05, **P<0.01.

As PARP1 suppresses LXRα transactivation through poly(ADP-ribosyl)ation, it was postulated that deletion of PARP1 will also lead to an increase in LXRα transactivity. However, knockdown of PARP1 dramatically suppressed the expression of LXRα target genes (Figure 4A). Concomitantly, PARP1 knockout inhibited expression of ABCG1 and ABCG5 (Figure 4B). These results clearly suggest that PARP1 is required for LXRα target expression. In support of this finding, forced expression of the human wild type PARP1 (wt-PARP1) restored ABCG1 and ABCG5 expression in PKO cells (Figure 4B).

We then examined the effects of PARP1 deficiency on the recruitment of LXRα to its target promoter. ChIP assays revealed that, PARP1 knockout decreased binding of LXRα to the *ABCG1* promoter. Since over-expression of wt-PARP1 restored this binding (Figure 4C), these data established that PARP1 was a prerequisite for LXRα binding to the target promoter. Besides, EMSA assays proved that PKO hepatocytes manifested decreased binding of the LXRα probe compared to WT hepatocytes (Figure 4D). Consistently, 3xLXRE driven luciferase activity was weaker in PKO cells than it was in WT cells (Figure 4E). Taken together, these data illustrate that PARP1 is required for binding of LXRα to LXRE in the target promoter and transactivation.

Now that it has been established that PARP1 is required for LXRα transactivation, we thus suspected that deficiency of PARP1 could aggravate HCD-induced cholesterol accumulation. As shown in Figure 4F, PKO mice displayed aggravated hepatic cholesterol accumulation, compared to their WT counterparts. Consistent with this, the expression of LXRα targets were significantly lower in PKO mice (Figure 4G). Intriguingly, despite PKO mice manifesting increased hepatic cholesterol accumulation, their hypercholesterolemia was ameliorated (Figure 4H-J), implying that the regulation of whole body cholesterol homeostasis by PARP1 is considerably more complicated than the current model.

In this study, the contrary effects of PARP1 deficiency and pharmacological inhibitors prompted us to speculate whether the protective effects of PJ34 and 3AB were mediated through PARP1. To confirm this, HCD fed PKO mice were treated with 3AB or PJ34. As shown in Figures 4K and 4L, the regulating effects of PJ34 and 3AB on HCD-induced hepatic cholesterol accumulation and LXRα target expression were abrogated. Consistently, PARP1 knockdown abolished PJ34-indcued ABCG1, ABCG5 and ApoE expression in HepG2 cells (Figure 4M). Taken together, the effects of 3AB or PJ34 on hepatic cholesterol accumulation and the LXRα pathway are mediated by PARP1.

### Un-poly(ADP-ribosyl)ated PARP1 promoted LXRα transactivation

**Figure 5:**
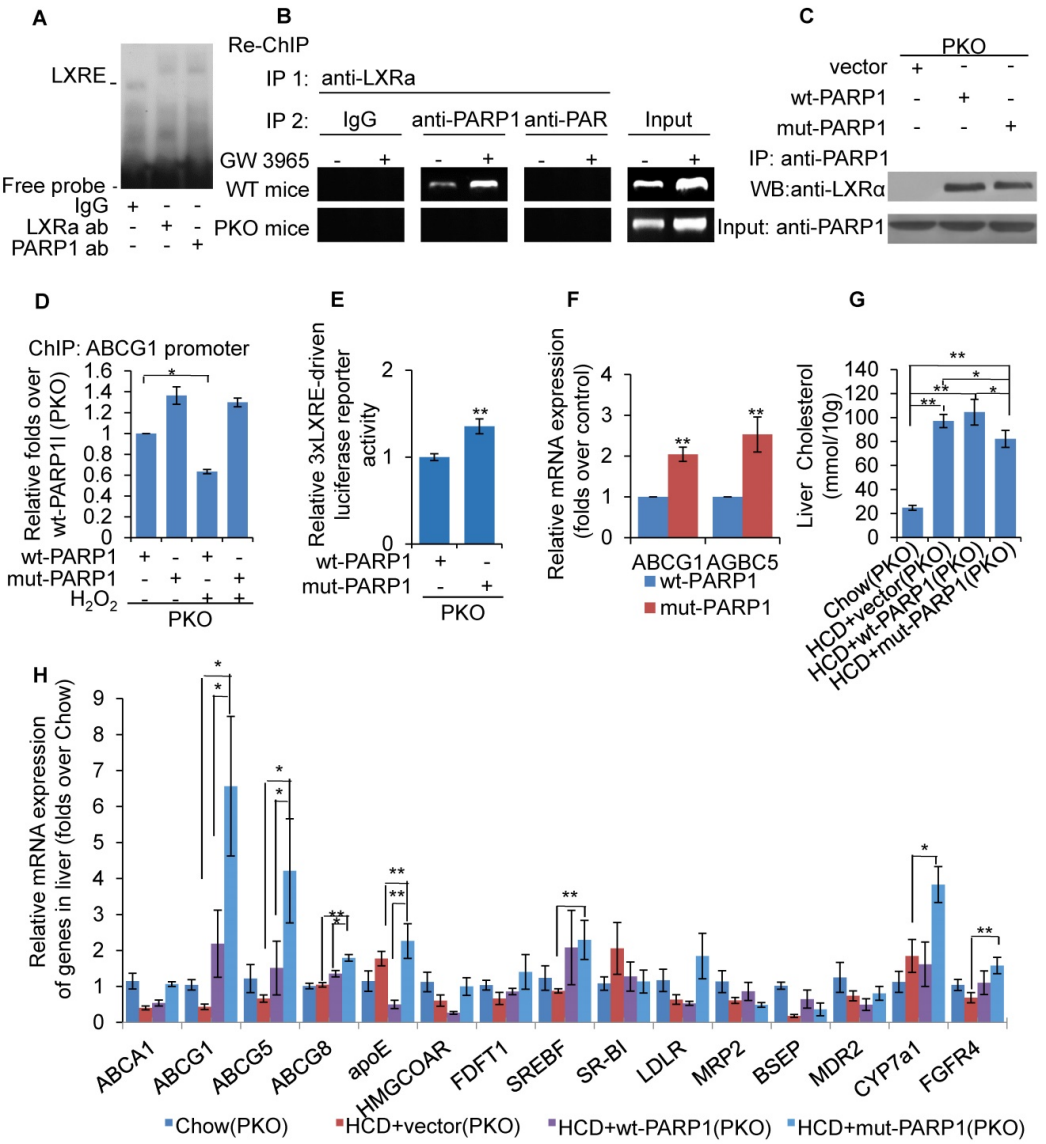
Unactivated (un-poly(ADP-ribosyl)ated) PARP1 promoted LXRα transactivation. (A) Nuclear extracts from hepatocytes from WT mice were incubated with anti-LXRα antibody, anti-PARP1 antibody or non-specific IgG (negative control), then analyzed by supershift assay (n=3). (B) re-ChIP assays, in which chromatin was first immunoprecipitated with anti-LXRα antibody then re-immunoprecipitated with anti-PARP1 antibody, or anti-PAR antibody (n=3). (C) Co-immunoprecipitation (Co-IP) of PARP1-bound proteins from hepatocytes of PKO mice, followed by Western blot analysis using anti-LXRα antibody. After transfection with full-length (wt-PARP1, 48h) or mutant (mut-PARP1, 48h) vectors, cells were treated with vehicle (PBS) or H_2_O_2_ (0.3 mM, 0.5h). (n=3). Data are expressed as mean ± SEM, ^*^P<0.05. (D) ChIP-PCR assay using anti-LXRα antibody for amplification of ABCG1 promoters in hepatocytes from PKO mice. Cells were transfected with a full-length (wt-PARP1) or mutant (mut-PARP1) vector for 48 hours (n=3). Data are expressed as mean ± SEM, *P<0.05. (E) mRNA expression of selected genes in hepatocytes from PKO mice transfected with a full-length (wt-PARP1) or mutant (mut-PARP1) vector for 48 hours (n=3). Data are expressed as mean ± SEM. **P<0.01 vs wt-PARP1 group. In G-H, PKO mice were fed a SCD (Chow, n=5) or HCD transfected with empty (n=5), full-length (wt-PARP1, n=5) or mutant (mut-PARP1, n=5) vector. (G) Cholesterol concentration in the livers of PKO mice. (H) mRNA expression of selected genes in PKO mice. In G-H, data are expressed as mean ± SEM, *P<0.05, **P<0.01.

As PARP1 is required for recruitment of LXRα to the target promoter, we suspected that it might be an important component of LXRα-LXRE complex. As shown in Figure 5A, incubation with anti-PARP1 antibody shifted the LXRα-LXRE complex band, indicating that PARP1 is indeed an essential component of the LXRα-LXRE complex. This finding was further confirmed by the results of the re-ChIP (ChIP on ChIP) assay, which revealed that PARP1 bound to ABCG1 promoter associated with LXRα (Figure 5B). Furthermore, the poly(ADP-ribosyl)ated protein could not bind to LXRE (Figure 5B), this indicated un-poly(ADP-ribosyl)ated, rather than activated, PARP1 was is required for the binding of LXRα to the ABCG1 promoter.

To further support this finding, the effects of mut-PARP1 and wt-PARP1 on the binding of LXRα to the ABCG1 promoter were investigated. Although lack of enzymatic activity did not alter the association between PARP1 and LXRα, forced expression of mut-PARP1 relative to wt-PARP1 resulted in clearly increased binding of LXRα to the ABCG1 promoter (Figures 5C and 5D). Moreover, treatment with H2O2 suppressed the wt-PARP1-induced, not mut-PARP1-induced, promoter binding of LXRα (Figure 5D). Taken together, these results indicate that unactivated/un-poly(ADP-ribosyl)ated PARP1 is a prerequisite for recruitment of LXRα to target promoter. Consistent with this, forced expression of mut-PARP1 resulted in higher luciferase activity driven by 3xLXRE than did wt-PARP1 transfection (Figure 5E), suggesting that unactivated PARP1 increased LXRα transactivity. Consistent with this finding, forced expression of mut-PARP1 relative to wt-PARP1 resulted in higher expression of the LXRα targets ABCG1 and ABCG5 in PKO cells (Figure 5F). Taken together, the results suggest that unactivated PARP1 promotes LXRα binding to its target promoter to increase gene transcription. Besides, forced expression of mut-PARP1 relative to wt-PARP1 or in cells transfected with empty vectors resulted in decreased cholesterol accumulation and enhanced hepatic expression of LXRα target genes involved in cholesterol disposal (Figures 5G & 5H). All these suggested that forced expression of mut-PARP1 might alleviate cholesterol accumulation through activation of LXRα signaling.

### Inhibition of PARP1-mediated ligand-induced LXRα transactivation

**Figure 6:**
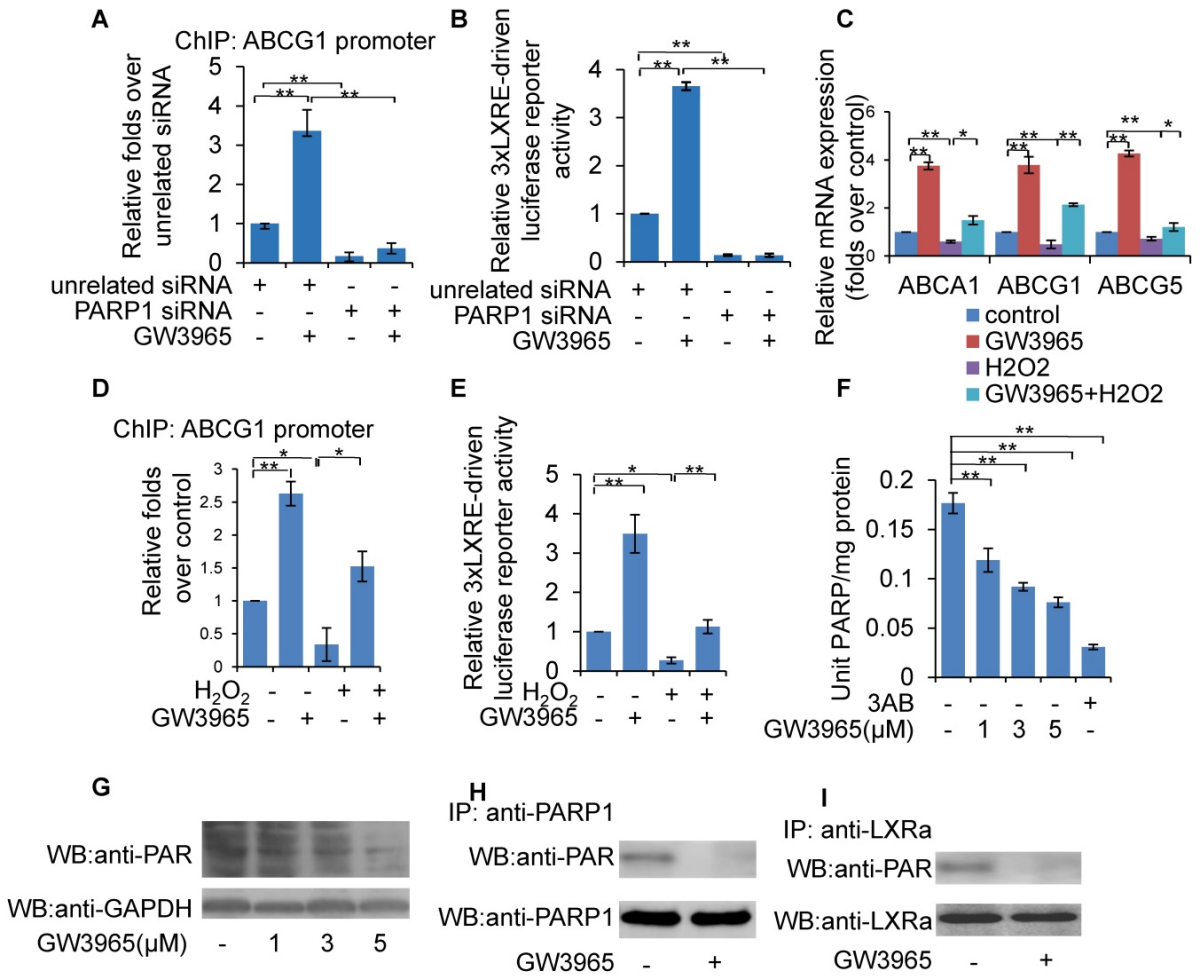
Inhibition of PARP1 mediated ligand-induced LXRα transactivation. Ligands included GW3965, T0901317 and 22(R)-hydroxycholesterol,. (A) ChIP-PCR assay using anti-LXRα antibody for amplification of ABCG1 promoters in HepG2 cells. Cells were treated with PARP1 siRNA (50 nM, 48h) or unrelated siRNA (50 nM, 48h) in the absence or presence of GW3965 (1 μM, 24h) (n=3). (B) Relative LXRE-driven luciferase activity in HepG2 cells. Cells were treated with PARP1 siRNA (50 nM, 48h) or unrelated siRNA (50 nM, 48h) in the absence or presence of GW3965 (1 μM, 24h) (n=3). (C) mRNA expression of selected genes in HepG2 cells treated with vehicle (PBS) or H_2_O_2_ (0.3 mM, 0.5h) in the absence or presence of GW3965 (1 μM, 24h) (n=3). (D) ChIP-PCR assay using anti-LXRα antibody for amplification of ABCG1 promoters in HepG2 cells treated with vehicle (PBS) or H_2_O_2_ (0.3 mM, 0.5h) in the absence or presence of GW3965 (1 μM, 24h) (n=3). (E) Relative LXRE-driven luciferase activity in HepG2 cells. Cells were treated with vehicle (PBS) or H_2_O_2_ (0.3 mM, 0.5h) in the absence or presence of GW3965 (1 μM, 24h) (n=3). (F) PARP activity in HepG2 cells. Cells were treated with GW3965 (1, 3, 5 μM), for 24 hours. 3AB (10 mM) was used as positive control (n=3). (G) Western blot assay of poly(ADP-ribosyl)ated proteins and PARP1 in whole extracts of HepG2 cells treated with vehicle (DMSO) or GW3965 (1, 3, 5 μM) (n=3). (H) Poly(ADP-ribosy)lation of PARP1 in HepG2 cells determined by IP with PARP1 followed by Western blot analysis using anti-PAR antibody. Western blotting with anti-PARP1 served as the loading control. Cells were treated with vehicle (DMSO) or GW3965 (1 μM, 24h) (n=3). (I) Poly(ADP-ribosyl)ation levels of LXRα in whole extracts of HepG2 cells determined by IP with LXRα followed by Western blot analysis using anti-PAR antibody. Western-blotting with anti-LXRα antibody served as the loading control. Cells were treated with vehicle (DMSO) or GW3965 (1 μM, 24h) (n=3). In A-F, data are expressed as mean ± SEM, *P<0.05, **P<0.01.

LXRα can be activated by endogenous oxysterols or the synthetic ligands GW3965 and T0901317. To determine whether PARP1 is involved in ligand-induced LXRα activation, PARP1 was knocked down using siRNA in HepG2 cells. As shown in Figure 6A, PARP1 knockdown abolished the recruitment of GW3965-induced LXRα to the ABCG1 promoter (Figure 6A). Consistent with this, knockdown of PARP1 also abrogated the GW3965-induced increase in 3xLXRE driven luciferase activity (Figure 6B). Taken together, PARP1 is a prerequisite for ligand-induced LXRα binding to its target promoter and transactivation.

In this study, activated PARP1 was shown to suppress LXRα transactivation through poly(ADP-ribosy)lation. We thus suspected that ligand-induced LXRα target expression might also be suppressed upon PARP1 activation. This hypothesis was confirmed by the observation that H_2_O_2_ treatment effectively suppressed GW3965-induced expression of ABCA1, ABCG1 and ABCG5 in HepG2 cells (Figure 6C). Consistent with this, GW3965-induced binding of LXRα to the ABCG1 promoter and increased 3xLXRE driven luciferase activity were also suppressed by H_2_O_2_ treatment (Figures 6D & 6E).

**Figure 7:**
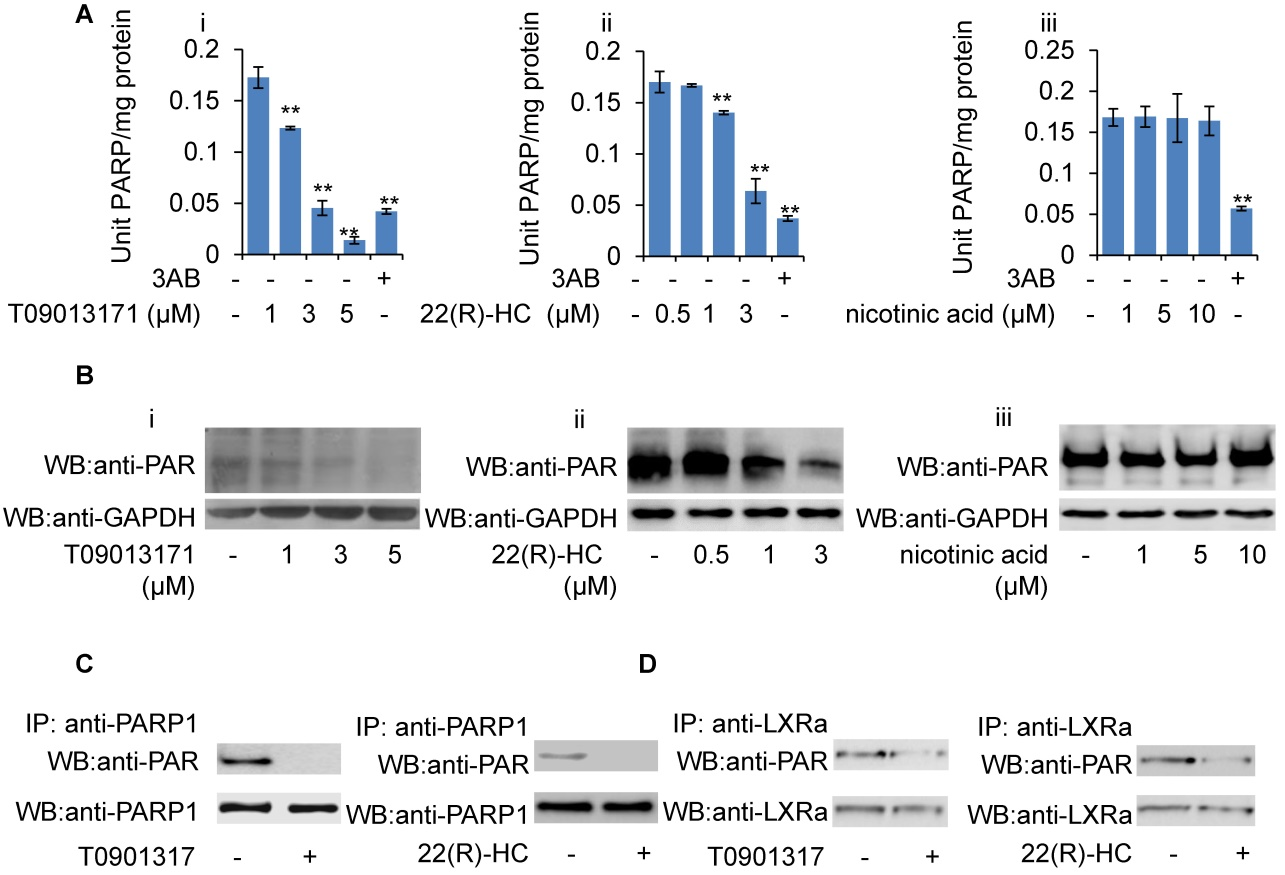
Role of T0901317 and 22(R)-HC on PARP activity. (A) mRNA expression of selected genes in HepG2 cells treated with PARP1 siRNA (50 nM, 48h) or unrelated siRNA (50 nM, 48h) in the absence or presence of T0901317 (1 μM, 24h) or 22(R)-HC (1 μM, 24h) (n=3). Data are expressed as mean ± SEM, ^*^P<0.05, ^**^P<0.01. (B) Western blot assay of poly(ADP-ribosyl)ated proteins in whole extracts of HepG2 cells treated with: (i) T0901317 (1, 3, 5 μM); (ii) 22(R)-hydroxycholesterol (0.5, 1, 3 μM) or (iii) nicotinic acid (1, 5, 10 μM) for 24 hours (n=3). (C) Poly(ADP-ribosy)lation of PARP1 in HepG2 cells determined by IP with PARP1 followed by Western blot analysis using anti-PAR antibody. Western-blotting with anti-PARP1 served as the loading control. Cells were treated with vehicle (DMSO), T0901317 (1 μM, 24h) or 22(R)-HC (1 μM, 24h) (n=3). (D) Poly(ADP-ribosyl)ation levels of LXRα in whole extracts of HepG2 cells determined by IP with LXRα followed by Western blot analysis using anti-PAR antibody. Western-blotting with anti-LXRα antibody served as the loading control. Cells were treated with vehicle (DMSO), T0901317 (1 μM, 24h) or 22(R)-HC (1 μM, 24h) (n=3).

Given that GW3965-induced LXRα transactivation could be suppressed by H_2_O_2_, together with the observation that PARP1 is a requirement for GW3965-induced LXRα transactivation, we suspected that these LXRα ligands might also suppress cellular PARP1 activity. HepG2 cells were treated with GW3965, T0901317 and 22(R)-HC, and the whole extracts were subjected to PARP activity assay and Western blot analysis. As shown in Figures 6F, 6G, 7A and 7B, administration of GW3965, T0901317 or 22(R)-HC reduced PARP activity and total protein poly(ADP-ribosy)lation levels in a dosage-dependent manner, indicating that cellular PARP activities were indeed suppressed.

In agreement with this, administration of GW3965 effectively suppressed poly(ADP-ribosy)lation of PARP1 (Figure 6H), indicating that GW3965 suppressed PARP1 activation. Accordingly, the poly(ADP-ribosy)lation of LXRα decreased significantly following GW3965 treatment (Figure 6I). Similar results were achieved when cells were treated with either T0901317 or 22(R)-HC (Figures 7C & 7D).

## Discussion

In this study, we revealed that activation of PARP1 promoted hepatic cholesterol accumulation through the impairment of LXRα signaling in liver when fed a HCD. Moreover, we identified LXRα as a substrate of the PARP1-mediated poly(ADP-ribosyl)ation reaction. Poly(ADP-ribosyl)ation prevents LXRα associating with its cognate target sequence (LXRE) in promoters, and thus suppresses gene transcription. Intriguingly, we found that unactivated PARP1 is an essential component of the LXRα-LXRE complex. Its deficiency inhibits LXRα binding to the target promoter, and thus suppresses gene transcription.

Long-term intracellular accumulation of free cholesterol in the liver can result in the formation of cholesterol crystals, potentially triggering liver inflammation 33. Therefore, liver cholesterol is regarded as a substantial risk factor for the development of non-alcoholic steatohepatitis (NASH) 34. As cholesterol homeostasis is maintained by the balance between cholesterol availability (intake and *de novo* synthesis) and disposal (catabolism and secretion), impaired LXRα signaling and resultant insufficiency of cholesterol disposal should inevitably lead to cholesterol over-accumulation, especially when challenged with high dietary cholesterol. In this study, suppression of PARP1 activation alleviates cholesterol accumulation by facilitating cholesterol disposal, indicating that PARP1-induced impairment of LXRα sensing is an important mechanism underlying liver cholesterol metabolic disorders, including NASH 20.

Poly(ADP-ribosyl)ation is an important post-translational modification of proteins. It is common for poly(ADP-ribosyl)ation to exert two contrary effects on the DNA-binding capacity of its acceptor proteins: suppression or promotion. The suppressive effects of protein-DNA interactions are usually attributed to the addition of negatively charged ADP-ribose polymer to acceptor proteins, resulting in a repulsion of the negative charges on the poly(ADP-ribosyl)ated modified proteins and DNA strands. Conversely, poly(ADP-ribosyl)ation can also increase the DNA binding capacity of many specific acceptor proteins, such as c-Jun and c-fos in the nucleus. This effect is generally mediated by the formation of a DNA-binding scaffold on the acceptor proteins, which increases the DNA binding capability of the acceptor proteins 20, 35. In this study, we found that poly(ADP-ribosyl)ation decreased the DNA binding activity of LXRα to LXRE, leading to decreased target gene transcription. Actually, we and others have previously demonstrated that poly(ADP-ribosyl)ation increases the DNA binding capacity of ERα, but decreases the capacity of PPARγ and PPARα 18, 19, 36. All these findings indicate that PARP1-mediated poly(ADP-ribosyl)ation is an important mechanism regulating the promoter binding and transactivity of many nuclear acceptors.

In the nucleus, LXRα can be activated by various ligands and synthetic agonists. Surprisingly, we found that inhibition of PARP1 activity mediates ligand-induced LXRα activation, including induction by GW3965, T0901317 and 22(R)-HC. From this finding it is rational to expect that an association with unactivated PARP1 is required for LXRα binding to the target promoter, either in the absence or presence of ligands. Moreover, although it is unknown whether this finding can be extended to all LXRα ligands, it at least suggests that inhibition of PARP1 activity might represent a novel strategy for the design of LXRα ligands/activators in the future.

In the present study, a very interesting finding is that, although PARP1 suppresses LXRα signaling through poly(ADP-ribosyl)ation, PARP1 *per se* is indispensable for LXRα transactivation and target expression. This is due to the essential role of unactivated PARP1 in the binding of LXRα to its target promoter, i.e. un-activated PARP1 is a transcriptional co-activator of LXRα. Indeed, the fact that PARP1 functions as a co-factor for transcription factors has been reported by many studies, e.g. Hassa et al. report that PARP1 is an important transcriptional co-factor of NF-κB, which promotes NF-κB transactivation through physical interaction 37. Interestingly, they also found that the enzymatic activity of PARP1 is unnecessary for its function as a transcriptional cofactor. However, in many conditions, poly(ADP-ribosyl)ation is an important mechanism regulating protein-protein interactions since it changes the structure of acceptor proteins by adding nucleic acid-like (poly(ADP-ribose)) polymer or forming a PAR-scaffold structure 38. However, according to our results, even though LXRα can bind to and interact with activated PARP1, and this binding inhibits target gene transcription.

The mechanism underlying the regulation of LXRα signaling is very complex and remains unclear to a large extent. A model has been proposed to describe the activation of LXRα in the nucleus: unligated LXRα binds to cognate LXREs and inhibits transcription by recruiting nuclear co-repressors. Ligand binding first results in dissociation of co-repressor and moderate activation of transcription. This then elicits recruitment of a co-activator, thus causing maximal activation of transcription 3, 39. However, our data revealed that, in the absence of ligands, some parts of LXRα are poly(ADP-ribosyl)ated by PARP1, which in turn, prevents recruitment to the target promoter. In this condition, LXRα signaling has not been activated. When ligands are present in the nucleus, PARP1 activity is suppressed, leading to increased nuclear content of unactivated PARP1 and un-poly(ADP-ribosyl)ated LXRα, which associate with each other and thereafter become recruited to the target promoters to facilitate gene transcription. This model takes the following findings into consideration: (1) LXRα can be poly(ADP-ribosyl)ated by PARP1 and poly(ADP-ribosyl)ation inhibits LXRα-promoter complex formation; (2) direct interaction with unactivated PARP1 is a prerequisite for the recruitment of LXRα to its target promoter; (3) LXRα ligands are PARP1 inhibitors and PARP1 is a prerequisite for ligand-induced LXRα transactivation. This model emphasizes the essential role of PARP1 in the regulation of the LXRα signaling pathway.

## Conclusions

Our study has revealed an important contribution of PARP1-mediation to the suppression of the LXRα signaling pathway in the pathogenesis of HCD-induced hepatic cholesterol over-accumulation. Furthermore, we have identified PARP1 as a key regulator of the LXRα signaling pathway, with inhibition of PARP1 activity mediating ligand-induced LXRα activation. These findings imply that PARP1 could be a potential target in the development of drugs and therapeutic strategies for the treatment of hepatic cholesterol metabolism disorders induced by impaired LXRα signaling.

## Supplementary Material

Supplementary figure S1.

## Figures and Tables

**Table 1:** Sequences of siRNAs used in this study

siRNA	Sense Sequence (5'-3')
PARP1	5'-GGAACAAGGATGAAGTGAA-3'
LXRα	5'-CACAGAGATCCGTCCACAA-3'
Unrelated siRNA	5'-UUCUCCGAACGUGUCACGU-3'

## Abbreviations

HCD: high cholesterol diet
KO: knockout
LXRα: Liver X receptor α
LXREs: LXR response elements
NRs: Nuclear receptors
PARP1: Poly(ADP-ribose) polymerase 1
RXR: retinoid X receptor
WT: Wildtype
