# Supplementary material for: Yin and Yang Regulation of Liver X Receptor α Signaling Control of Cholesterol Metabolism by Poly(ADP-ribose) polymerase 1
Source: Int J Biol Sci. 2020 Sep 1;16(15):2868–82. doi: 10.7150/ijbs.50042 (PMC7545717; doi:10.7150/ijbs.50042)
Supplement: Supplementary file 1 — Supplementary figure S1. [file ijbsv16p2868s1.pdf]

Figure S1. (A) mRNA expression of PARP1 in HepG2 cells transfected with empty (pCDNA3.1), full-length (wt-PARP1) or mut-PARP1 vector for 48 hours. (n=3). (B) mRNA expression of LXRα in HepG2 cells. Cells were pretreated with LXRα siRNA (50 nM, 48h) or unrelated siRNA (50 nM, 48h) (n=3). Data are expressed as mean ± SEM, $^{**}P<0.01$.

Fig. S1

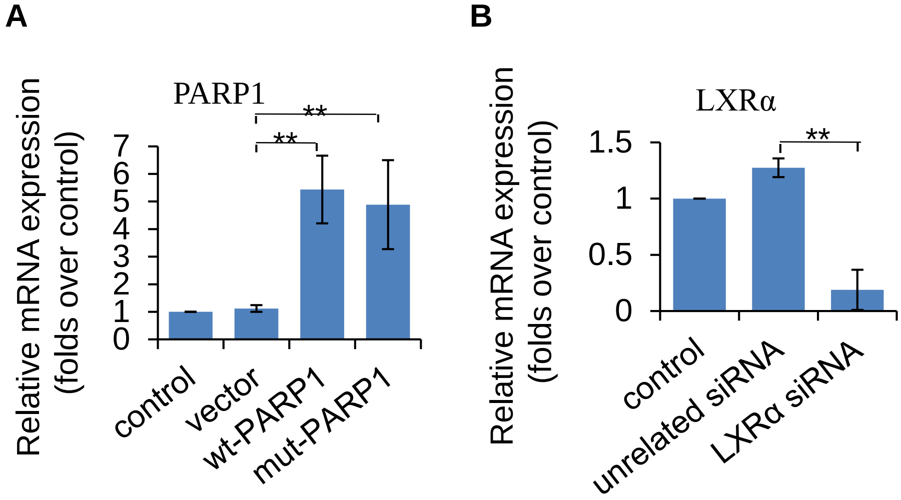
A
PARP1
Relative mRNA expression
(folds over control)
**
**
7
6
5
4
3
2
1
0
control
vector
wt-PARP1
mut-PARP1
B
LXRα
Relative mRNA expression
(folds over control)
**
1.5
1
0.5
0
control
unrelated siRNA
LXRα siRNA
